# Differing needs for Advance Care Planning in the Veterans Health Administration: use of latent class analysis to identify subgroups to enhance Advance Care Planning via Group Visits for veterans

**DOI:** 10.1186/s12910-024-01117-w

**Published:** 2024-10-28

**Authors:** Monica M. Matthieu, Songthip T. Ounpraseuth, J. Silas Williams, Bo Hu, David A. Adkins, Ciara M. Oliver, Laura D. Taylor, Jane Ann McCullough, Mary J. Mallory, Ian D. Smith, Jack H. Suarez, Kimberly K. Garner

**Affiliations:** 1 U.S. Department of Veterans Affairs Medical Center, Central Arkansas Veterans Healthcare System, HSR&D Center of Innovation: Center for Mental Healthcare & Outcomes Research, 2200 Fort Roots Dr. North Little Rock, Arkansas, 72114 USA; 2 School of Social Work, Saint Louis University, Saint Louis, Missouri USA; 3 Fay W. Boozman College of Public Health, University of Arkansas for Medical Sciences, Little Rock, Arkansas, USA; 4 U.S. Department of Veterans Affairs Medical Center, Central Arkansas Veterans Healthcare System , Geriatric Research, Education and Clinical Center, Little Rock, Arkansas, USA; 5 College of Medicine, Department of Psychiatry, University of Arkansas for Medical Sciences, Little Rock, Arkansas, USA

**Keywords:** Advance care planning, Veterans, Veterans Health Administration, Latent class analysis, Advance directives

## Abstract

**Background:**

Advance Care Planning via Group Visits (ACP-GV) is a patient-centered intervention facilitated by a clinician using a group modality to promote healthcare decision-making among veterans. Participants in the group document a “Next Step” to use in planning for their future care needs. The next step may include documentation of preferences in an advance directive, discussing plans with family, or anything else to fulfill their ACP needs. This evaluation seeks to determine whether there are identifiable subgroups of group participants with differing needs prior to delivery of the ACP-GV program and, if so, to use information about the subgroups to enhance the program offered to veterans in United States Department of Veterans Affairs (VA) healthcare settings.

**Methods:**

We conducted a secondary analysis of national data from a quality improvement evaluation. Patient- and provider-level data from administrative healthcare records for VA users in all 50 states, territories, and the District of Columbia provides data on veterans attending ACP-GV during federal fiscal years 2018–2022 (*N* = 26,857). Latent class analysis seeks to identify the various subgroups of veterans based on their level of ACP self-efficacy before attending ACP-GV and any demographic differences across the resulting subgroups of veterans attending ACP-GV. ACP self-efficacy is derived from seven items obtained from a participant worksheet used during the group.

**Results:**

Analysis revealed two distinct groups of veterans, distinguishable by their pre-ACP-GV levels of one aspect of ACP self-efficacy: prior knowledge of ACP. Veterans with higher prior knowledge of ACP are associated with an identified next step focused on checking their current AD status and updating it, and veterans with lower ACP prior knowledge are associated with identifying a next step to discuss ACP more fully with family. Differences in age, sex, race, ethnicity, and marital status exist across subgroups of veterans.

**Conclusion:**

Greater attention must be paid to ACP and veterans’ prior knowledge of ACP to consistently encourage annual review and status updates.

## Introduction

In the United States Department of Veterans Affairs (VA), Advance Care Planning (ACP) is an ongoing clinical priority to ensure veterans’ health care aligns with their values and treatment preferences [1]. An ACP discussion allows veterans to identify their care preferences for use during a time when they are no longer capable of communicating or making decisions [2]. Studies support a variety of positive outcomes across process, action, quality of care [3, 4], and health status domains [5] related to ACP.

VA, as a cabinet level federal department, operates three separate entities; the Veterans Health Administration (VHA) provides healthcare services, the Veterans Benefits Administration provides benefits, and the National Cemetery Service provides burial and internment services. In VHA, Advance Care Planning via Group Visits (ACP-GV) is a patient-centered intervention facilitated by a clinician in a group setting with other veterans and those they trust. The participants, along with a group facilitator, discuss the key aspects of healthcare decision-making, and the facilitator encourages a “Next Step” following ACP discussion to elicit behavioral intentions for future action. An optional next step is completing an Advance Directive (AD), which is a written legal document that records the veteran’s care wishes to guide the receipt of their future and desired health care. The AD also includes a mental healthcare preferences section for participants at-risk of or living with a mental health condition.

Despite the public health significance [6] and national legislation [7] supporting the need to offer ACP to patients in healthcare settings, ACP for all veterans seeking health care in VA has not yet garnered the desired results. Previous literature estimates the rate of AD discussions (defined as any engagement with veterans about ACP individually or in a group visit) among VA healthcare users in federal fiscal year (FY) 2020 as 5.19% which reached 49,824 veterans [8], far less than the target goal of 9.07 million veteran beneficiaries enrolled in VHA [9]. Moreover, the goal of reducing age, race, ethnicity, and sex disparities for a range of ACP outcomes [6] in VHA, such as care consistent with goals or accessibility to documents and recorded wishes when needed, also remains unrealized. Compared to those who had no AD discussion documented (individual or group), veterans in the VHA AD discussion cohort were middle aged (54 to 74 years), African American, males, living in urban areas, and had an established VA disability rating (i.e., “Priority Group 1–4”) [8]. In order to optimize health care for veterans and align it with their individualized preferences, goals, and values, ACP is critically needed to reach all veterans, regardless of their health or other statuses, who seek VA health care.

## What is advance care planning via group visits?

ACP-GV promotes healthcare decision-making and communication with veterans and those they trust, in-person or virtually, using an educational format led by a trained ACP-GV facilitator. The group lasts 60 min and is offered for up to ten participants to discuss educational topics related to ACP. The group format includes group facilitation, review of materials, and discussion of key concepts to include ACP definitions, optional aspects of an AD, and reminders to annually review the AD. First, using best practice strategies of motivational interviewing [10], the facilitator encourages participation in conversation, eliciting life experiences and active discussion among the participants instead of didactic presentation of material by the instructor to the participants. Next, in addition to these topics and tasks, the facilitator reviews specific materials, such as an ACP-GV Participant Worksheet, which gauges prior knowledge and experience with ACP and ADs. The participant worksheet is also used at the end of group to query knowledge gained from the group. Lastly, participants are encouraged to set a next step which elicits personalized behavioral intentions for future action [11]. The participant defines their next step as a result of a goal-setting activity, which is also offered at the end of the group. Examples of next steps include communicating care decisions to family, caregivers, trusted others, or the healthcare team if they are incapacitated (e.g., unconscious, on a ventilator, or cognitively impaired) or the completion of an AD to guide healthcare decision-making for the veteran. These individual next steps are developed by the veteran and then shared with the ACP-GV facilitator who enter the type of next step into the healthcare record. The use of an additional tool, the ACP-GV Fidelity Instrument, orders the tasks and topics, guides the facilitator through the educational content, and is used to self-assess or peer rate the facilitator’s adherence to the ACP-GV model. By utilizing aspects of motivational interviewing in a group setting, ACP-GV provides an opportunity for active engagement and conversation with other veterans, caregivers, and those they trust [12].

## Background on ACP self-efficacy guiding this latent class analysis

The foundation for the work that we report here include topics broadly conceptualized in the literature as the elements of self-efficacy, or the participants’ own belief in their ability to make a behavior change [13]. These elements include exploring apparent heterogeneity in the participant population to attend ACP-GV based on knowledge, confidence, comfort, outcome expectancies, and motivation. For heuristic purposes and potential practical application, we examine whether there are distinct subgroups among veterans who report the intention to take a next step by the end of the group. The next step is a classification given for a range of actions that is categorically selected and coded into the healthcare record by the ACP-GV facilitator based on data provided by the veteran at the end of the group on the participant worksheet. We focus on veteran-centered factors related to this behavioral activation to define our subgroups of veterans and assess the above self-efficacy factors applied to ACP, collectively referred to as “ACP self-efficacy” [14]. Behavioral activation is a methodology that encourages veterans to distinguish their values, anticipate barriers to ACP, and plan for a future activity (next step) [15]. Behavioral activation may include an additional review of the content and process of executing an AD or the completion of a written AD as a next step following participation in ACP-GV.

We chose ACP self-efficacy to frame our knowledge questions for ACP-GV participants for three reasons; first, despite the robust relationship between ACP knowledge, self-efficacy, and actions needed to complete an AD, there is a paucity of research identifying how these factors may cluster into subgroups of individuals likely to complete an AD. Completing an AD is defined herein as a process or set of steps to actively prepare a written document specifying preferences, whereas a complete AD is the possession of an active, current, written, and fully executable legal document to guide future health care. Second, there are a growing number of evidence-based programs that increase access to and engagement in ACP, especially for those veterans living in rural areas [16]. Basic epidemiological data about subgroups of veterans with this co-occurring ACP self-efficacy and a gap in completion of the AD document can help to inform and increase the number of ACP programs available for veterans and those they trust. Third, lack of an AD in its own right is associated with high morbidity and mortality that separately and collectively pose significant public health burdens [17]. Understanding how these ACP self-efficacy items co-occur among veterans who have attended ACP-GV provides opportunities for intervention approaches to reduce potentially negative outcomes among veterans that an AD might mitigate.

Prior ACP knowledge is highlighted in this study; nearly all military service members have experience with ADs, as it is a requirement to complete an AD prior to deployment. Furthermore, similarly to VHA, within Department of Defense, ACP is briefed as part of the process for initial enrollment in healthcare benefits and is a mandated screening in clinic settings for all military service members who receive health care. Therefore, all veterans have prior knowledge of ACP from their service in the U.S. Armed Forces; however, the recall of that information is variable and may potentially contribute to lower prior knowledge on the ACP-GV Participant Worksheet.

Using secondary data from a national quality improvement evaluation of the delivery of ACP-GV to veterans through VHA, we examine whether there are identifiable subgroups of ACP-GV participants with differing needs going into the group. Based on the experience of our clinical partners who note differences in the types of veterans based on the responses to initial questions related to ACP self-efficacy, our findings about the subgroups will be used for program enhancements. Our analyses have three stages. First, using latent class analysis (LCA), we identify how knowledge, confidence, comfort, outcome expectancies, and motivation as indicators of ACP self-efficacy co-occur in subgroups of veterans who indicate on the participant worksheet wanting to take a next step following attendance at ACP-GV delivered in VHA settings. Second, we explore the demographic characteristics of these subgroups––based upon demographic variables such as marital status, race, and sex–– potentially providing vital data needed to appropriately shape ACP initiatives tailored for different veteran subpopulations. Lastly, we associate each type of next step with class membership while accounting for potential demographic differences. A next step is a powerful predictor of behavioral activation leading to action, which in ACP can be the decision to communicate with a trusted other, care team, or to complete an AD. In summary, the aim of this evaluation is to determine whether there are identifiable subgroups of group participants with differing needs prior to delivery of the ACP-GV program and, if so, to use information about the subgroups to enhance the program offered to veterans in VA healthcare settings.

## Methods

### Study design

We conducted a secondary analysis of national data collected for a quality improvement evaluation of the delivery of ACP to veterans using a group modality within VA healthcare settings. LCA seeks to identify the various subgroups of veterans based on their level of ACP self-efficacy before attending ACP-GV and any demographic differences using age, marital status, sex, Hispanic ethnicity, race, rurality, and VHA priority group across the resulting subgroups of veterans.

### Data sources and study setting

ACP-GV is a patient-centered intervention facilitated by a clinician in a group modality to promote healthcare decision-making with veterans, their caregivers, and/or trusted others.

### Data collection/extraction methods

We use patient- and provider-level data from the Corporate Data Warehouse (CDW) which houses administrative healthcare records for VHA service users in all 50 states, territories, and the District of Columbia. Specifically, the sample (*N* = 26,857) includes unique veterans who received ACP-GV, where unique means that veterans are counted only once using the index date of their first ACP-GV in a federal FY. Attendance in ACP-GV is specifically documented using a four-digit character code (i.e., CHAR 4 code) in the electronic health record used in VHA. Therefore, these data represent unique veterans attending ACP-GV once per year using the index date across five federal FY (2018–2022). We did not use data prior to FY 2018 due to inconsistencies in the data; FY 2018 also coincides with the emergence of the CHAR 4 code indicating participation specifically in ACP-GV.

## Measures

### Age, marital status, sex, hispanic ethnicity, race, rurality, VHA priority group

Categorical variables are available in the CDW for veteran related demographic characteristics, rurality, and VHA priority group for each FY, which are described previously [8]. For this evaluation, VA administrative data provides an adequate sample size for subgroup analysis by mean age, marital status (i.e., divorced, married, not married, or separated), race (i.e., Black or African American, White or Caucasian, other races), male or female sex, Hispanic or not Hispanic ethnicity, and rurality used by VA to categorize veteran’s residences as rural or urban using VA administrative data and VHA priority group status. VHA priority group status is healthcare eligibility defined by group numbers and descriptions for the inclusion criteria for each group. While a general description is provided here, more details can be found elsewhere [18]. Priority 1–4 are veterans who have a VA service-connected disability, receive VA aid and attendance, receive VA homebound services, or who are determined to be catastrophically disabled; Priority 5–6 are veterans who do not have a service-connected disability, have a non-compensable service-connected disability that VA rated as 0% disabling and have an annual income level that is below VA adjusted income limits (based on resident zip code), receive VA pension benefits, or are eligible for Medicaid programs (group 5) or have a non-compensable service-connected disability that VA rated as 0% disabling and meet criteria of certain military related exposures or war zones. Priority 7–8 are veterans whose income is above (group 8) or below (group 7) VA income limits and geographically adjusted income limits for where they live and who agree to copayments. See Table 1 for the description of the total sample.

### Latent class indicators

We used seven items of ACP self-efficacy derived from the ACP-GV Participant Worksheet used in ACP-GV. The participant worksheet was created by the National ACP-GV Program provided within VHA to all facilities with special attention devoted to veterans residing in rural areas. The first seven questions are reviewed and answered by participants at the start of the group session (e.g., pre-intervention). Knowledge is measured with one question, written as “How knowledgeable are you about advance care planning?” with a five-item Likert response option: “not at all, a little bit, moderately, quite a bit, and extremely.” Comfort is assessed with two questions: “I have thought about what I would want if I were hurt, injured or sick and could not communicate” and “I have thought about my treatment preferences if I could not communicate them during a mental health episode.” Response options for these and subsequent questions were “Yes” or “No.” Confidence is assessed with four items: “I have talked with someone I trust to make health care decisions for me; I have named someone to make health care decisions for me; I have discussed these topics with someone on my health care team (such as a doctor, nurse, or social worker); I have filled out an advance directive (also known as ‘living will’) to guide those I trust to make health care decisions for me.” We used the first seven items in the LCA to identify veteran subgroups and assign class membership to each veteran in our sample. Table 2 displays the question, response options, and patterns for each of the seven latent class indicators.

### Additional ACP self-efficacy measures and outcome expectancy items

Near the end of the ACP-GV discussion, participants return to the worksheet and write responses to a final set of questions (Questions 8–11). A post-intervention knowledge gain question is queried first with “How much has your knowledge increased about advance care planning?” with a five-item Likert response option (i.e., not at all, a little bit, moderately, quite a bit, extremely). For outcome expectancies, the participants are asked, “If you are ready to take a next step” and instructed to enter their next step in the blank text box, if applicable. To ease administrative burden for providers, the National ACP-GV Program scripted a range of possible categorical response options within the electronic health record documentation to include: [1] Check current AD status and update as needed [2], Complete AD [3], Discuss with family [4], Discuss with health care provider [5], Discuss with non-family member [6], File a copy of an existing AD [7], Goals not clear/unsure [8], Learn more about ACP [9], Update existing AD [10], I need time to think and reflect on my values and wishes, and [11] Other that are used to document the next step response. Responses are documented and referred to in a follow up phone contact with the veteran to engage them in their next step.

The worksheet then provides four text boxes to reflect on the development of a goal if a next step was identified previously. The question prompt reads “If you are ready to take a next step, please write what your next step will be,” followed by four questions with their own respective text box, “When will you do this?”, Who will you do this with?”, How will you do this?”, Where will you do this?” The final question related to the likelihood of taking the next step, or behavioral activation, is queried with “How likely are you to take this next step?” with a five-item Likert response (i.e., definitely will not, probably will not, not sure, probably, definitely will). See Table 2 for a description of the types of next steps and the distribution of the types of next steps by the resulting class membership.

### Statistical analysis

#### Latent class analysis

To identify subgroups of veterans’ knowledge, confidence, and comfort with ACP, we conducted a series of LCA using robust maximum likelihood estimation with the MPlus version 8.8 software [19]. We used a combination of criteria to determine the number of latent classes, including (1) examination of fit indices (e.g., Akaike Information Criteria [AIC] and Bayesian Information Criteria [BIC], etc.) of which we weighed the values for the BIC (smaller is better) and the sample adjusted BIC as most accurate given its superior performance for LCA models, (2) Vuong-Lo-Mendell-Rubin Likelihood Ratio Test (VLMR-LRT) where significant p-value indicates the (K-1) class model is rejected in favor of a model with at least k-classes, (3) entropy values (larger is better), and (4) clinical judgment regarding the practical utility of classes for intervention efforts [20]. Consistent with the views of Nylund et al. (2007) [20], we first identify the point where our model fit indices start to plateau across the different LCA models we executed. This flattening effect suggests minimal or no improvement in model fit with the inclusion of additional classes. To decide whether to include additional classes after fit indices values start to plateau, we consider the heuristic and clinical value of adding additional classes and weighed this against the value of using more parsimonious solutions. We note that veterans did not provide responses to all items of the ACP self-efficacy questionnaire; therefore, the LCA analysis was based on case-wise deletion.

#### Comparison across subgroups

After identifying the best fitting LCA model and assigning each veteran to the classes with highest membership probability, descriptive measures, including frequency and percentages, describe veteran demographic characteristics overall and are stratified by latent classes. Additionally, demographic characteristics across the latent classes are compared using chi-square tests (see Table 1). For each next step response category, separate logistic regression models access the association between the latent class member and types of next steps taken. In addition, multivariable logistic regression accounts for demographic characteristics including age, marital status, race, ethnicity, urban/rural, sex, and VHA priority group. We report both unadjusted and adjusted odds ratios (OR) along with their respective 95% confidence intervals (CI).

## Results

### Number and description of subgroups of veterans

After inspection of the values for the fit indices, we compared the differences between the two- and three-class solutions using the AIC and BIC. BIC and sample-size adjusted BIC for the two-class solution resulted in a corresponding entropy rating of 0.76 compared to entropy rating of 0.73 for the three-class model. While the VLMR-LRT had a p-value < 0.0001 for the three-classes model, suggesting that three classes is an improvement over the two-classes model, based on the gain in entropy and trade-off in model complexity, we opt for the two-classes model based on clinically relevant interpretable reasons. The average individual veteran posterior probabilities for being assigned to a specific latent class matrix has high values along the diagonal (92.7% and 93.3%, respectively) indicating good classification. Finally, out of 23,020 veterans included in the analysis, 10,952 (48%) are categorized as class one while 12,068 (53%) are categorized as class two.

### Overall sample characteristics

Table 1 describes veterans enrolled in VHA who participated in ACP-GV during federal FY 2018–2022 (*N* = 26,857). The mean age was nearly 60 years of age (SD = 14.66), predominately married (50%), two-thirds Caucasian (66%), and the vast majority were non-Hispanic (95%) males (86%). Most of the veterans who participated in ACP-GV during this time frame were from urban areas (64%) and were in VHA priority group 1–4 (74%), indicating veterans with a service-connected disability rating.

**Table 1:** Demographics and class membership based on prior ACP knowledge for veterans enrolled in VHA who participated in ACP-GV during the federal FY of 2018–2022 (*N* = 26,857)

Demographics of Veterans Attending ACP-GV in FY 18–22		Total number of ACP-GV participants		Class 1: Higher Prior ACP Knowledge		Class 2: Lower Prior ACP Knowledge		*P* value
		*N* = 26, 857^a^		*n* = 10,952^b^		*n* = 12,068^b^		
Age, mean ± SD		59.29 ± 14.66		61.55 ± 14.10		57.34 ± 14.84		< 0.0001
Marital Status	Divorced	7,328	29.09%	2,877	26.70%	3,515	29.62%	< 0.0001
	Married	12,686	50.36%	5,597	51.94%	5,109	43.06%	
	Not married	5,177	20.55%	1,838	17.06%	2,655	22.38%	
	Separated	1,170	4.64%	464	4.31%	586	4.94%	
Race *	Black or African American	7,578	30.47%	2,951	28.76%	3,443	30.71%	< 0.0001
	White or Caucasian	16,441	66.12%	6,987	68.09%	7,348	65.54%	
	Other races	848	3.41%	324	3.16%	420	3.75%	
Ethnicity	Not Hispanic or Latino	22,742	95.03%	9,600	95.29%	10,120	94.39%	< 0.0001
	Hispanic or Latino	1,190	4.97%	474	4.71%	602	5.61%	
Rural/Urban	Rural	9,775	36.48%	4,105	37.53%	4,333	35.97%	< 0.0001
	Urban	17,018	63.52%	6,832	62.47%	7,714	64.03%	
Sex	Female	3,693	13.75%	1,513	13.81%	1,517	12.57%	< 0.0001
	Male	23,164	86.25%	9,439	86.19%	10,551	87.43%	
VHA Priority Group**	Priority 1–4	19,490	73.66%	8,141	75.44%	8,483	71.11%	< 0.0001
	Priority 5–6	6,094	23.03%	2,281	21.14%	3,063	25.67%	
	Priority 7–8	877	3.31%	370	3.43%	384	3.22%	

*Note* This analysis utilized VA administrative data from federal fiscal year 2018–2022, which is prior to and during the VA’s efforts to utilize more inclusive language in data collection based on the Department of Veterans Affairs Inclusion, Diversity, Equity, & Access (I-DEA) Action Plan September 2021 https://www.va.gov/ORMDI/docs/VA_I-DEA_Action_Plan-SIGNED.pdf

*Other races utilized for data analysis by the U.S Government include American Indian, Alaskan Native, Asian, Native Hawaiian, and Other Pacific Islander

**VHA Priority Groups are defined as: Priority 1–4 are veterans who have a VA disability; Priority 5–6 are veterans who have a 0% VA disability, VA pension, or annual income lower than VA adjusted income levels, select military service before 1988; Priority 7–8 are veterans whose income is above and below the VA income limits and geographically adjusted income limits for where they live, and agree to copayments. For more detail, see: https://www.va.gov/health-care/eligibility/priority-groups/. ^a^The sample size 26,857 represents the total number of veterans enrolled in VHA who participated in ACP-GV during federal FY 2018–2022;^b^ Not everyone responded to each question; therefore, those with missing observations were not included in the latent class analysis. The sample sizes for Class 1 and 2 were 10,952 and 12,068, respectively, which does not sum to the total sample of 26,857

### Subgroups of veterans and ACp self-efficacy indicators

Using the first seven elements of ACP self-efficacy listed in Table 2, which were queried before participating in ACP-GV, we identified the various subgroups of veterans. For the two-class solution, we observed a propensity for veterans to endorse different levels of knowledge based on veterans who responded to question one, “How knowledgeable are you about advance care planning?” with a five-item Likert response. In particular, 10% of veterans in class one endorse the response “not at all,” whereas 35% of veterans in class 2 endorse that response. Based on the response pattern among veterans, we label class one veterans as “higher prior ACP knowledge” and class two as “lower prior ACP knowledge”.

**Table 2:** ACP questions and class membership based on prior ACP knowledge for veterans enrolled in VHA who participated in ACP-GV during fiscal years of 2018–2022 (*N* = 26,857)

Questions and Responses at Beginning of ACP-GV		Total number of ACP-GV participants (*N* = 26,857)^a^		Class 1: Higher Prior ACP Knowledge (*n* = 10,952)^b^		Class 2: Lower Prior ACP Knowledge (*n* = 12,068)^b^	
Q1: How knowledgeable are you about advance care planning	**Responses**,** N (%)**						
	Not at all	5,386	20.05%	1,124	10.26%	4,262	35.32%
	A little bit	6,960	25.92%	2,747	25.08%	4,213	34.91%
	Moderately	4,845	18.04%	2,859	26.10%	1,986	16.46%
	Quite a bit	2,784	10.37%	2,429	22.18%	355	2.94%
	Extremely	782	2.91%	702	6.41%	80	0.66%
	Missing/No response	6,100	22.71%	1,091	9.96%	1,172	9.71%
Q2: I have thought about what I would want if I were hurt, injured or sick and could not communicate:	Yes	16,418	61.13%	10,280	93.86%	6,138	50.86%
	No	5,155	19.19%	300	2.74%	4,855	40.23%
	Missing/No response	5,284	19.68%	372	3.40%	1,075	8.91%
Q3: I have thought about my treatment preferences if I could not communicate them during a mental health episode:	Yes	11,411	42.49%	7,865	71.81%	3,546	29.38%
	No	8,578	31.94%	1,771	16.17%	6,807	56.41%
	Missing/No response	6,868	25.58%	1,316	12.02%	1,715	14.21%
Q4: I have talked with someone I trust to make healthcare decisions for me;	Yes	13,984	52.07%	10,231	93.42%	3,753	31.1%
	No	7,487	27.88%	225	2.05%	7,262	60.18%
	Missing/No response	5,386	20.06%	496	4.53%	1,053	8.73%
Q5: I have named someone to make healthcare decisions for me:	Yes	11,357	42.29%	9,765	89.16%	1,592	13.19%
	No	10,442	38.88%	772	7.05%	9,670	80.13%
	Missing/No response	5,058	18.84%	415	3.79%	806	6.68%
Q6: I have discussed these topics with someone on my healthcare team (such as a doctor, nurse or social worker);	Yes	7,094	26.41%	5,622	51.33%	1,472	12.2%
	No	14,487	53.94%	4,636	42.33%	9,851	81.63%
	Missing/No response	5,276	19.64%	694	6.34%	745	6.18%
Q7: I have filled out an advance directive (also known as ‘living will’) to guide those I trust to make healthcare decisions for me;	Yes	6,933	25.81%	6,362	58.09%	571	4.73%
	No	14,909	55.51%	4,179	38.16%	10,730	88.91%
	Missing/No response	5,015	18.68%	411	3.75%	767	6.36%
**Questions and responses to be completed by the participant at end of ACP-GV**							
Q8: How much has your knowledge increased about advance care planning?;	Not at all	549	2.04%	274	2.5%	272	2.25%
	A little bit	1,047	3.9%	530	4.84%	509	4.22%
	Moderately	3,994	14.87%	1,724	15.74%	2,251	18.65%
	Quite a bit	9,002	33.52%	4,188	38.24%	4,789	39.68%
	Extremely	3,711	13.82%	1,932	17.64%	1,763	14.61%
	Missing/No Response	8,554	31.85%	2,304	21.03%	2,484	20.59%
Q11: How likely are you to take this next step?;	Definitely will not	320	1.19%	174	1.59%	139	1.15%
	Probably will not	220	0.82%	79	0.72%	139	1.15%
	Not sure	893	3.33%	250	2.28%	633	5.25%
	Probably	3,090	11.51%	1,146	10.46%	1,922	15.93%
	Definitely will	6,948	25.87%	3,536	32.29%	3,347	27.73%
	Missing/No Response	15,386	57.29%	5,767	52.65%	5,888	48.79%

*Note*^a^The sample size 26,857 represents the total number of veterans enrolled in VHA who participated in ACP-GV during federal FY 2018–2022;^b^ Not everyone responded to each question; therefore, those with missing observations were not included in the latent class analysis. The sample sizes for Class 1 and 2 were 10,952 and 12,068, respectively, which does not sum to the total sample of 26,857

Upon identifying the two latent classes, we examined the veteran demographics across the classes (see Table 1). Veterans assigned to class one (higher prior ACP knowledge) were older with an average age of 62 years of age compared to 57 years of age among those assigned to class two (lower prior ACP knowledge). Veterans in class one were also more likely to be married (51.9% vs. 43.1%; *p* < 0.0001) and had a higher proportion of Caucasian veterans (68.1% vs. 65.5%; *p* < 0.0001). Class one had a slightly higher proportion of veterans from rural areas (37.5% vs. 35.9%; *p* < 0.0001) and slightly more women (13.8% vs. 12.6%; *p* < 0.0001). Finally, class one, veterans with higher prior ACP knowledge, had more veterans in the Priority 1–4 category (75.4% vs. 71.1%; *p* < 0.0001).

### Outcome expectancies: rates of veterans taking the next step

Differences and odds ratios for the type of next steps differentiated between both veterans in class one (higher prior knowledge; the reference group) and class two (lower prior knowledge) are in Table 3. First, across both latent class subgroups of the total sample, the type of next step following ACP-GV is completing an AD (26.79% vs. 27.70%) and discussing with a family member (17.45% vs. 27.67%), respectively. After adjusting for key demographic characteristics, the odds of discussing with family as the next step among veterans with lower prior knowledge (class two) are 1.90 (95% CI: 1.77, 2.03; *p* < 0.0001) times the odds for veterans with higher prior knowledge (class one). In other words, the odds of choosing to discuss with family as the next step among veterans with lower prior knowledge is 77% higher than the odds for veterans with higher prior knowledge. Similarly, the odds of identifying the next step of completing an AD for the lower prior knowledge veterans are 14% higher than the odds for veterans with higher prior knowledge (adjusted OR = 1.14; 95% CI: 1.07, 1.21; *p* < 0.0001). However, the greatest significant difference between the classes is observed with checking current AD status and updating as needed with 17.52% of veterans in class one (higher prior knowledge of ACP) compared to 1.47% in class two (lower prior knowledge of ACP) (adjOR = 0.07; 95% CI: 0.06, 0.09).

**Table 3:** Number and types of next steps coded by clinical providers that VHA enrolled veterans plan to take following participation in ACP-GV during the federal fiscal years of 2018–2022

Type of Next Step*, *N* (%)	Class 1: Higher ACP Prior Knowledge Responses (*n* = 10,952)	Class 2: Lower ACP Prior Knowledge Responses (*n* = 12,068)	Unadjusted Odds Ratio (95% CI)^a^	Adjusted Odds Ratio (95% CI)^a,^**
Complete an advance directive	2,934 (26.79)	3,343 (27.70)	1.05 (0.99, 1.11)	1.14 (1.07, 1.21)
Check current advance directive status and update as needed	1,919 (17.52)	177 (1.47)	0.07 (0.06, 0.08)	0.07 (0.06, 0.09)
Update existing advance directive	483 (4.41)	42 (0.35)	0.08 (0.06, 0.10)	0.08 (0.05, 0.11)
File a copy of an existing r	418 (3.82)	14 (0.12)	0.03 (0.02, 0.05)	0.03 (0.02, 0.06)
Discuss with family	1,911 (17.45)	3,339 (27.67)	1.81 (1.70, 1.93)	1.90 (1.77, 2.03)
Discuss with non-family member	55 (0.50)	170 (1.41)	2.83 (2.09, 3.84)	2.49 (1.79, 3.47)
Discuss with healthcare provider	123 (1.12)	111 (0.92)	0.82 (0.63, 1.06)	0.83 (0.63, 1.11)
Learn more about advance care planning	144 (1.31)	561 (4.65)	3.66 (3.04, 4.40)	3.60 (2.95, 4.38)
I need time to think and reflect on my values and wishes.	99 (0.90)	383 (3.17)	3.59 (2.88, 4.49)	3.53 (2.74, 4.54)
Goals not clear/unsure	181 (1.65)	432 (3.58)	2.21 (1.85, 2.63)	2.00 (1.65, 2.41)
Other	1,181 (10.78)	1,404 (11.63)	1.09 (1.00, 1.18)	1.09 (1.00, 1.20)

*Note* *Types of next steps are coded by VHA providers who are facilitating ACP-GV. Veterans complete a fill in the blank text box on the worksheet for Question 9 before the end of group. When documenting participation in the electronic health record, providers review the veterans’ written responses and select as many of these categories from a list that apply to the veterans written response. These data reflect the veterans who were included in the LCA analyses, which uses case wise deletion (*N* = 23,020). **Multivariable logistic regression model adjusted for demographic characteristics including age, marital status, race, ethnicity, rural/urban, sex, and VHA priority. ^a^Class 1 (higher prior knowledge) is the reference group

The odds of wanting to learn more about ACP as their next step among veterans with lower prior knowledge was nearly four times the odds for veterans with higher prior knowledge (adjOR = 3.60; 95% CI: 2.95, 4.38; *p* < 0.0001), likewise for needing time to think and reflect on values and wishes (adjOR = 3.53; 95%: CI = 2.74, 4.54; *p* < 0.0001). In other words, choosing to learn more about ACP as the next step was nearly 260% higher among veterans with lower prior knowledge compared to veterans with higher prior knowledge. Similarly significant findings are noted for reflecting on values and wishes.

**Table 4:** Demographics and class membership of veterans who participated in ACP-GV and who also indicated taking a next step among during the federal FY of 2018–2022 (*N* = 10,038)

Demographics of Veterans Attending ACP-GV and Identified a Next Step		Number of Veterans Attending ACP-GV and Identified a Next Step		Class 1: Higher ACP Prior Knowledge		Class 2: Lower ACP Prior Knowledge		*P* value
		*N* = 10,038		*n* = 4,682		*n* = 5,269		
Age, mean ± SD		58.57 ± 15.05		60.67 ± 14.47		56.75 ± 15.31		< 0.0001
Marital Status	Divorced	2,726	27.16%	1,212	25.89%	1,490	28.28%	< 0.0001
	Married	4,775	47.57%	2,428	51.86%	2,311	43.86%	
	Not married	1,906	18.99%	749	16.00%	1,132	21.48%	
	Separated	408	4.06%	195	4.16%	213	4.04%	
	Unknown	223	2.22%	98	2.09%	123	2.33%	
Race	Black or African American	2,372	23.63%	1,033	22.06%	1,316	24.98%	< 0.0001
	White	6,618	65.93%	3,214	68.65%	3,353	63.64%	
	Other races	329	3.28%	130	2.78%	196	3.72%	
	Unknown	719	7.16%	305	6.51%	404	7.67%	
Ethnicity	Not Hispanic or Latino	8,213	81.82%	3,957	84.52%	4,189	79.5%	< 0.0001
	Hispanic or Latino	442	4.4%	194	4.14%	244	4.63%	
	Unknown	1,383	13.78%	531	11.34%	836	15.87%	
Rural Urban	Rural	3,479	34.66%	1,669	35.65%	1,794	34.05%	0.0080
	Urban	6,532	65.07%	3,002	64.12%	3,459	65.65%	
	Unknown	27	0.27%	11	0.23%	16	0.30%	
Sex	Female	1,461	14.55%	709	15.14%	726	13.78%	< 0.0001
	Male	8,577	85.45%	3,973	84.86%	4,543	86.22%	
Priority	Priority 1–4	7,188	71.61%	3,454	73.77%	3,667	69.60%	0.0001
	Priority 5–6	2,309	23.00%	974	20.8%	1,318	25.01%	
	Priority 7–8	377	3.76%	174	3.72%	201	3.81%	
	Unknown	164	1.63%	80	1.71%	83	1.58%	

*Note* *VHA Priority Groups are defined as: Priority 1–4 are veterans who have a VA disability; Priority 5–6 are veterans who have a 0% VA disability, VA pension, or annual income lower than VA adjusted income levels, select military service before 1988; Priority 7–8 are veterans whose income is above and below the VA income limits and geographically adjusted income limits for where they live, and agree to copayments. For more detail, see: https://www.va.gov/health-care/eligibility/priority-groups/. *N* = 10,038 is the number of veterans who participated in ACP-GV who also indicated who took a next step (i.e., responses of probably and definitely will take a next step. Missing data was handled by case wise deletion

### Demographic differences across the subgroups of veterans intending to take a next step

Statistically significant differences are noted in the demographics of veterans who specified a possible next step and indicated being probably or definitely likely to take the step following ACP-GV participation (*N* = 10,038). Across the two subgroups of veterans, there are more veterans (52.5%) in class two, reflecting veterans with lower prior knowledge who intend to take a next step than veterans with higher prior knowledge in class one (46.6%). There are also statistically significant differences in age, sex, race, ethnicity, and marital status found across subgroups. Namely, veterans with higher prior knowledge of ACP (class one) who indicated intending to take a next step after attending the group were older, female, Caucasian, not Hispanic/Latino, and married compared to veterans in the lower prior knowledge (class two). The age difference comparing ages between sub-groups of participants who identified a next step and indicated that they would probably/definitely take the step was notable, with veterans with higher prior knowledge being significantly older (M = 60.67; SD ± 14.47) than veterans with lower prior knowledge (M = 56.75; SD ± 15.31) (see Table 4).

## Discussion

The aim of this LCA is to identify subgroups of veterans who identify a next step or intend to take a next step following ACP-GV by examining indicators of ACP self-efficacy, identifying demographic characteristics across the subgroups of veterans, and associating each type of next step with class membership while accounting for potential demographic differences. Findings reveal two distinct groups of veterans, distinguishable by their pre-ACP-GV levels of one aspect of ACP self-efficacy: knowledge. Demographically, veterans with higher prior ACP knowledge were older, married, Caucasian, female, living in rural areas, and had more priority disabled veterans than those veterans in class two who reported lower prior knowledge of ACP.

In terms of next steps, both classes have a high propensity for endorsing wanting to discuss with family as their intended next step following participation in ACP-GV. However, the odds of wanting to learn more about ACP as their intended next step among veterans with lower prior knowledge of ACP were nearly four times the odds for veterans with higher prior knowledge (OR = 3.850; CI = 3.199–4.634). Age, sex, race, ethnicity, and marital status differences are noted across subgroups of veterans intending to take a next step; veterans with higher prior knowledge of ACP (class one) who intend to take a next step after attending the group were older, Caucasian, female, not Hispanic/Latino, and married compared to veterans with lower prior knowledge (class two).

### Prior knowledge of ACP

We return to the idea that prior ACP knowledge is important for many veterans who may have engaged in these discussions while in the military, while others were required to complete an AD prior to deployment. As confirmed by the LCA, all seven indicators are greater for the higher knowledge class, indicating that most veterans not only have prior knowledge, but many also noted their next step was completing or updating an existing AD. Yet, due to systemic electronic health record storage issues [8], it is unknown how many veterans in our sample actually had an existing or complete AD.

### Outcome expectancies: Next steps matter

In terms of outcomes, one finding is consistent with the program which is to regularly review an existing and complete AD and to update it accordingly as life events change. Clearly, changes in health (both physical and mental), cognitive functioning, and relationship status occur across the life course, so ACP-GV emphasizes the following message to all veterans, caregivers, and those they trust, “Things change, so too does your Advance Directive!” Findings related to discussing preferences and healthcare wishes with families is also a hallmark of ACP-GV, where national partnerships with the Caregiver Support Program and Blind Rehabilitation Services are increasing the level of family and caregiver involvement and adaptions for accessibility. Finally, how to make and message the priority of discussion with the healthcare team is an ongoing challenge especially relevant in VHA’s movement to be an Age-Friendly healthcare system [21] that emphasizes what matters most to veterans.

### Implications for practice and policy

Implications for clinical practice center around improvements in training for clinicians and dissemination of the National ACP-GV Program. We begin with specific recommendations for ACP-GV facilitators and those conducting AD conversations with veterans. First, when recruiting veterans to participate in ACP-GV, it may be beneficial to note that ACP is not new for many veterans, and ACP may have been offered during their military experience. Additionally, asking about prior knowledge and experience with ACP is a standard talking point in ACP-GV when reviewing the participant worksheet and eliciting personal experiences. From this discussion and responses to the worksheet, facilitators can anticipate how to more meaningfully guide discussions about setting a next step. Finally, clinicians, equipped with the findings from this study, can encourage those with significant prior knowledge of ACP, who may be more inclined to take a next step, to update and revise their AD, while encouraging those with limited prior knowledge to talk with family members. In summary, our findings support clinicians in a variety of ways in recruiting and engaging veterans, caregivers, and/or those they trust in ACP-GV.

As a quality improvement project, these results can also guide our partners to focus on the implications of higher and lower knowledge of ACP before the launch of any local program. This is because previous knowledge of ACP among veterans can support the formation of groups offered in different settings and to different subgroups of veterans. One use of this data our partners note is to enhance existing training materials for new ACP-GV staff. The training currently focuses on how the group facilitator can structure the group and review the participant worksheet to learn more about the participants. Inclusion of these findings support the perceived differences among participants already noted by experienced ACP-GV facilitators.

Policy efforts to ensure that all healthcare systems screen for ADs is part of a mandate and a Joint Commission requirement. VHA complies with the Patient Self Determination Act of 1990 by ensuring that all beneficiaries are screened for presence of an AD [7]. However, the quality of these screening interactions is debatable. Much has been written of late regarding the outcomes of ACP, with outcomes such as cost and return on investment the focus more so than the quality of the interaction [22]. Annual updating of ADs can serve an important reminder to connect with these requirements. Additionally helpful for healthcare executives is to note that access to ACP, choice, and veteran preferences are honored when ACP is offered individually, in groups, and/or using telehealth for those facilities with an active ACP-GV program.

### Limitations

We are mindful to consider potential limitations of our data, so any conclusions we draw are viewed cautiously. LCA as an analytic strategy provides a heuristic and clinical profile of veterans; however, it does not provide any further contextualization beyond the measures and indicators included. LCA is limited and relies on clinical expertise in the phenomena studied. Therefore, important variables may have been overlooked. Second, the ACP self-efficacy measures were developed for clinical purposes with an in-person delivery and paper and pencil self-administered materials to guide the progression of content discussed in group. While required, it is possible that missing data occurred for a variety of reasons, to include veteran refusal and provider mishandling or loss of paperwork. Next, VHA administrative data for ACP-GV requires clinician data entry; thus, the type of next steps that veterans endorsed in this data may be biased due to data entry errors. Some challenges also remain with virtual modalities, including completion/distribution of the materials and provider data entry. Hard copies of the participant worksheet provide data for this report; however, challenges remain in using the worksheet in virtual modalities of ACP-GV. Finally, we note that results do not generalize to veterans who have not attended ACP-GV, those who do not complete a worksheet, or veterans who complete ACP individually in VHA settings.

## Conclusions

Our evaluation is unique in that it uses a national sample of veterans enrolled in VHA who attended ACP-GV across five federal FY to identify heuristic and clinically relevant subgroups of veterans. These data are based on their answers to ACP self-efficacy questions on a participant worksheet used in the group. We found two subgroups; first, veterans with higher prior knowledge of ACP are associated with an identified next step focused on checking their current AD status and updating it. Second, veterans with lower ACP prior knowledge are associated with identifying a next step to discuss ACP more fully with family. Greater attention must be paid to ACP and veterans’ prior knowledge of ACP to consistently encourage annual review and status updates.

Our evaluation indicates that patient-centered ACP interventions delivered in groups of veterans, caregivers, and those they trust remain an important engagement goal for all veterans. While veterans have an awareness of ACP from their time in the military, attending a group with other veterans may provide a refresher of that knowledge which may motivate them to complete or update an AD in VHA.

## Data Availability

All raw data are the property of the United States Government; data availability will be subject to review by the Department of Veterans Affairs and must be in compliance with all applicable federal policies and laws.
